# Induction of Neuroinflammation and Brain Oxidative Stress by Brain-Derived Extracellular Vesicles from Hypertensive Rats

**DOI:** 10.3390/antiox13030328

**Published:** 2024-03-07

**Authors:** Xinqian Chen, Xin Yan, Leah Gingerich, Qing-Hui Chen, Lanrong Bi, Zhiying Shan

**Affiliations:** 1 Department of Kinesiology and Integrative Physiology, Michigan Technological University, Houghton, MI 49931, USA; 2 Health Research Institute, Michigan Technological University, Houghton, MI 49931, USA; 3 Department of Chemistry, Michigan Technological University, Houghton, MI 49931, USA

**Keywords:** neuroinflammation, oxidative stress, extracellular vesicles, hypertension, reactive oxygen species (ROS)

## Abstract

Neuroinflammation and brain oxidative stress are recognized as significant contributors to hypertension including salt sensitive hypertension. Extracellular vesicles (EVs) play an essential role in intercellular communication in various situations, including physiological and pathological ones. Based on this evidence, we hypothesized that EVs derived from the brains of hypertensive rats with salt sensitivity could trigger neuroinflammation and oxidative stress during hypertension development. To test this hypothesis, we compared the impact of EVs isolated from the brains of hypertensive Dahl Salt-Sensitive rats (DSS) and normotensive Sprague Dawley (SD) rats on inflammatory factors and mitochondrial reactive oxygen species (mtROS) production in primary neuronal cultures and brain cardiovascular relevant regions, including the hypothalamic paraventricular nucleus (PVN) and lamina terminalis (LT). We found that brain-derived DSS-EVs significantly increased the mRNA levels of proinflammatory cytokines (PICs) and chemokines, including TNFα, IL1β, CCL2, CCL5, and CCL12, as well as the transcriptional factor NF-κB in neuronal cultures. DSS-EVs also induced oxidative stress in neuronal cultures, as evidenced by elevated NADPH oxidase subunit CYBA coding gene mRNA levels and persistent mtROS elevation. When DSS-EVs were injected into the brains of normal SD rats, the mRNA levels of PICs, chemokines, and the chronic neuronal activity marker FOSL1 were significantly increased in the PVN and LT. Furthermore, DSS-EVs caused mtROS elevation in brain PVN and LT, particularly in neurons. Our study reveals a novel role for brain-derived EVs from hypertensive rats in triggering neuroinflammation, upregulating chemokine expression, and inducing excessive ROS production. These findings provide insight into the complex interactions between EVs and hypertension-associated processes, offering potential therapeutic targets for hypertension-linked neurological complications.

## 1. Introduction

Extracellular vesicles (EVs) are pivotal conduits for intercellular communication, profoundly influencing multicellular organisms’ physiological and pathological processes [1]. These vesicles, encompassing both exosomes and macrovesicles, serve as cellular couriers, shuttling complex cargoes of proteins, lipids, metabolites, and various nucleic acids, including mRNA, microRNA, and long non-coding RNA [2]. The molecular payload, encapsulated within the protective lipid bilayer of EVs, is meticulously tailored to reflect the donor cells’ health and activity status, thereby transferring materials and functional information [2,3]. The targeting of specific recipient cells allows EVs to deliver signals that can alter gene expression, modulate cellular behavior, and orchestrate a coordinated response among cells in diverse biological contexts [2,3]. With their inherent ability to navigate through the extracellular milieu and deliver their cargo precisely, EVs are increasingly recognized as important players in maintaining homeostasis and influencing the progression of diseases [4,5,6,7], marking them as a focal point for therapeutic innovation and a deeper understanding of cellular communication networks.

Hypertension, a critical risk factor for cardiovascular diseases [8,9], is influenced by a complex interplay of factors, notably high dietary salt intake [10]. Elevated salt intake activates immune responses and sparks inflammatory processes in key brain regions associated with cardiovascular control, such as the hypothalamic paraventricular nucleus (PVN) [11,12]. The ensuing neuroinflammation disrupts neurotransmitter balance critical for blood pressure (BP) regulation, contributing to the pathogenesis of hypertension [13,14]. This condition is further exacerbated by inflammatory mediators like cytokines and chemokines, which emerge from salt-induced neuroinflammation, heightening sympathetic nervous system activity (SNA) and reducing baroreflex sensitivity, thus fostering hypertension [14,15,16,17]. A pivotal consequence of increased salt consumption is the surge in the generation of reactive oxygen species (ROS) within the brain, straining the brain’s antioxidant defenses and leading to oxidative stress. This oxidative stress is a critical perpetrator in the cascade of events that exacerbate hypertension, as it activates pro-inflammatory pathways and boosts SNA [18,19,20,21,22], which are recognized contributors to elevated BP [23]. The lamina terminalis (LT), with its constituent structures—the subfornical organ, the organum vasculosum of the LT, and the median preoptic nucleus—and its lack of a complete blood–brain barrier (BBB), is particularly susceptible to circulating factors such as ions, hormones, and cytokines. It is crucial in body fluid homeostasis and cardiovascular regulation [24,25]. It has been observed that pro-inflammatory cytokines are elevated in the LT of rats subjected to a high-fat diet and Ang II infusion, highlighting the significance of the cytokine-mediated impairment of LT functions and the overactivation of its neural circuitry during hypertensive conditions [26].

Based on compelling evidence, we hypothesize that EVs originating from the brains of hypertensive rats contribute to neuroinflammation and oxidative stress, playing a role in the onset of hypertension. To test our hypothesis, we examined the impact of brain derived EVs from hypertensive Dahl Salt-Sensitive (DSS) rats on neuroinflammation and oxidative stress, comparing them to EVs from normotensive Sprague Dawley (SD) rats. Specifically, we investigated how these EVs influence cytokine and chemokine levels and ROS and other oxidative stress indicators in both neuronal cultures and specific brain regions linked to BP control. Our study may uncover new targets for high BP treatment.

## 2. Materials and Methods

### 2.1. Animals

Adult SD and DSS rats were obtained from Charles River Laboratories (Wilmington, MA, USA) and utilized in our breeding colony to generate offspring. The rats were housed under controlled conditions, with an ambient temperature ranging from 20 to 24 °C and a 12 h light–dark cycle. The rats were provided ad libitum access to food and water throughout the experimental period. The rats were used for intracerebroventricular (ICV) injection, brain-derived EV isolation, and primary neuronal cell preparation; all procedures are described below. All experimental protocols were approved by the Institutional Animal Care and Use Committee (IACUC) at Michigan Technological University.

### 2.2. Isolation of Brain-Derived EVs

Six-week-old male DSS rats were given a high-salt diet (4% NaCl) to develop hypertension [11], while age-matched SD rats received a regular salt diet (0.4% NaCl) as a control group. Following a minimum six-week dietary regimen, the animals were euthanized for brain extraction. The whole brain, except for the cerebellum, was used to isolate EVs using a revised established protocol [27,28]. Brain tissues were sectioned into small pieces and incubated in Gibco Hibernate E medium at a ratio of approximately 0.2 g of tissue to 2 mL of medium per well in a 6-well plate. The tissue was then dissociated to a uniform size of about 2 × 2 × 2 mm. To this, collagenase D and DNase I were introduced to achieve concentrations of 2 mg/mL and 40 U/mL, respectively, in each well. The plate was incubated at 37 °C for 30 min with gentle shaking at 70 rpm. Post incubation, protease and phosphatase inhibitors were added before the tissue was strained through a 70 μm cell strainer into a new 50 mL tube. Subsequent differential centrifugation steps were conducted at 4 °C (300× *g* for 10 min, 2000× *g* for 20 min, and 10,000× *g* for 30 min). The resulting supernatants were passed through a 0.22 μm filter to exclude larger particles. These filtered supernatants were overlaid onto a 30% sucrose cushion and ultracentrifuged at 100,000× *g* for 90 min at 4 °C. Pellets were collected and washed with Dulbecco’s phosphate-buffered saline (DPBS) with ultracentrifugation at 100,000× *g* for 90 min again at 4 °C. The final pellets obtained were resuspended in 50 μL of ice-cold DPBS, enhanced with protease and phosphatase inhibitors, to prepare for downstream applications.

### 2.3. Scanning Transmission Electron Microscopy (STEM) Analysis

EVs in a 30 μL aliquot were combined with an identical volume of 2% paraformaldehyde (PFA) and left to interact for 5 min. Then, 5 μL of this mixture was placed onto the carbon-coated side of a grid and allowed to settle for 1 min. The grid was then blotted gently to dry using filter paper. For negative staining, uranyl acetate (UA) was employed. Freshly prepared 2% UA solution, 3 μL in volume, was applied to the grid and allowed to remain for 30 s before blotting away the excess solution with filter paper. After repeating the staining procedure, the grid was left to dry in the air overnight. The dry samples were subsequently examined under a FEI 200 kV Titan Themis STEM.

### 2.4. Dynamic Light Scattering (DLS) Analysis

For particle size determination, a 10 μL volume of the resuspended EV pellet was diluted in DPBS to a protein concentration of 0.01 μg/μL. Approximately 800 μL of this dilution was placed into a low-volume cuvette. The Malvern Zetasizer Nano series was utilized to assess the size distribution of the particles. Cuvettes with lids were inverted before measurements to ensure uniformity and prevent the sedimentation of larger particles. Each dilution was analyzed in triplicate to ensure the accuracy of the results.

### 2.5. Western Blot Analysis

The presence of specific proteins in EVs was evaluated through Western blot analysis. The EVs were prepared by mixing with RIPA lysis buffer containing 0.5% phenylmethylsulfonyl fluoride (PMSF) and subjected to sonication in three 5 s bursts. The mixture was then incubated on ice for 15 min, pipetting at 5 min intervals to assist lysis. Protein concentrations were quantified using the Bradford reagent. Proteins (40 to 100 μg) were then separated on an SDS-PAGE gel using an electric field (initially 80 mV for 30 min, followed by 120 mV for 1.5 to 2.5 h). After electrophoresis, proteins were transferred to a nitrocellulose membrane with a trans-blot turbo transfer system. The membrane was blocked with 5% milk in Tris-buffered saline containing 0.1% Tween^®^ 20 (TBST) and then incubated with primary antibodies including mouse anti-ALG-2 interacting protein X (mouse-anti-Alix, 1:200 dilution, Santa Cruz Biotechnology, Dallas, TX, USA), mouse anti-tumor susceptibility 101 (mouse anti-TSG101, 1:200 dilution, Santa Cruz Biotechnology), mouse anti-Golgi matrix protein 130 kD (mouse anti-GM130, 1:200 dilution, Santa Cruz Biotechnology), and rabbit anti-CD9 (1:1000 dilution, Cell Signaling, Danvers, MA, USA) at 4 °C overnight. The membranes were washed thrice with PBS, each for 5 min, followed by an hour’s incubation with horseradish peroxidase (HRP)-conjugated secondary anti-bodies. Chemiluminescent detection was achieved using SuperSignal West Dura Extended Duration Substrate and imaged using a Bio-Rad gel imaging system. Primary antibodies are listed in Table S1.

### 2.6. Fluorescent Labeling of Brain-Derived EVs

Following established protocols, brain-derived EVs were labeled with rhodamine-based fluorophores [29]. The labeled EVs (Rho-EVs) underwent incubation with primary neuronal cultures. The uptake and localization of these Rho-EVs were examined at intervals of 3, 24, 48, and 72 h using confocal microscopy (Olympus FV1000, Waltham, MA, USA). Hoechst stain (0.1 g/mL) was applied to the neuronal cultures 10–15 min before imaging to visualize cell nuclei. To track EV distribution in vivo, Rho-EVs were injected into the right lateral ventricle of SD rats. Twenty-four h post EV administration, rats were euthanized with an overdose of isoflurane, followed by transcranial perfusion with 4% PFA. Subsequently, peripheral organs, including the adrenal gland, heart, intestine, kidney, liver, and spleen, were collected and sectioned. Tissue cell nuclei were stained with 4′,6-diamidino-2-phenylindole dihydrochloride (DAPI). The distribution of EVs and their co-localization with tissue cells were then observed under a confocal microscope.

### 2.7. Intracerebroventricular (ICV) Injections of EVs

The procedures for ICV injections adhered to previously published protocols [30,31]. Rats under anesthesia with 2.5% isoflurane in O_2_ received an injection of 8 µg of EV protein from either DSS or SD rats into the right lateral ventricle. Stereotaxic coordinates guided the injections: 0.8–0.9 mm posterior to bregma, 1.4–1.8 mm lateral to the midline, and 3.2–3.8 mm below the dural surface. Injections were administered at a flow rate of 1 µL/min using an UltraMicroPump3 (World Precision Instruments, Sarasota, FL, USA). Following the procedure, the animals were euthanized with an excess of isoflurane. Their brains were immediately frozen in liquid nitrogen and stored at −80 °C for subsequent mRNA analysis.

### 2.8. mRNA Level Measurement Using Real-Time PCR

The measurement of mRNA levels for select genes was performed in neuronal cultures and specific brain regions (PVN and LT areas) utilizing real-time PCR, as detailed in earlier studies [30,31]. RNA was isolated using the RNeasy Mini kit as per the manufacturer’s protocol. Between 200 and 400 ng of RNA from each sample was reverse-transcribed to synthesize cDNA, which was then used as a template in real-time PCR assays. The expression levels of cytokines including tumor necrosis factor alpha (TNFα), interleukin-1 beta (IL-1β), and interleukin 6 (IL-6), chemokines including C-C motif chemokine ligand 2 (CCL2), CCL5, and CCL12, and the nuclear factor NF-kB subunit NF-kB1, inducible nitric oxide synthase (iNOS), NADPH oxidase subunits cytochrome b-245, alpha polypeptide, and beta polypeptide (CYBA and CYBB), as well as indicators of neuronal activity including FBJ osteosarcoma oncogene and Fos-like antigen 1 (c-FOS and FOSL1), were quantified. TaqMan-specific primers and probes were employed for these assessments, with glyceraldehyde-3-phosphate dehydrogenase (GAPDH) mRNA as a normalization reference. PCR primers were obtained from Thermo Fisher Scientific, and the detailed primer information, along with the accession numbers of the corresponding genes, is provided in Supplementary Table S1.

### 2.9. Mitochondrial Reactive Oxygen Species (mtROS) Measurement

Mitochondria-targeting fluorescent probes (MitoProbe, Waltham, MA, USA) [32] were employed to determine ROS levels in the mitochondria of primary brain neuronal cultures and brain tissue. During in vitro experiments, primary neurons, aged 7 to 10 days in culture, were incubated with MitoProbe (1 μM) after either a direct application or a fixation with 4% PFA for 30 min, followed by a co-stained with Hoechst for 15 min. After incubation, the cells were cleansed with PBS and analyzed using confocal microscopy to evaluate mtROS levels.

For in vivo measurements, rats received ICV injections of EVs. Three hours after the EV administration, MitoProbe was injected into the same brain region. Six hours after the initial EV injections, an overdose of isoflurane was administered for euthanasia, followed by transcardial perfusion with cold PBS and then 4% PFA in 1 × PBS. The brains were then extracted, fixed in 4% PFA overnight, and preserved in 30% sucrose in 1 × PBS until the tissue descended to the container’s bottom. Subsequently, the brains were cryo-sectioned for immunofluorescence analysis.

### 2.10. Immunofluorescence Analysis

The process for immunofluorescence began with brains being embedded in O.C.T. compound (Sakura Finetek, Dallas, TX, USA) and then cryo-sectioned into 20 µm thick coronal sections that included the PVN and LT regions. Following established protocols, brain sections were initially rinsed three times in PBS for 10 min each, permeabilized in cold methanol for 10 min, and then given a PBS wash. The sections were subsequently blocked with 5% horse serum for one hour. Incubation followed with primary antibodies: rabbit anti-neuronal nuclei (rabbit anti-NeuN, 1:300 dilution), mouse anti-glial fibrillary acidic protein (mouse anti-GFAP, 1:300 dilution), or rabbit anti-ionized calcium binding adaptor molecule 1 (rabbit anti-Iba1, 1:500 dilution) in PBS containing 0.5% Triton X-100 and 5% horse serum, maintained for 24 h at 4 °C. On the following day, the sections underwent three further PBS washes, each lasting 10 min, before incubation with secondary antibodies—Alexa Fluor 488 donkey anti-rabbit IgG or Alexa Fluor 488 donkey anti-mouse IgG—for one hour at room temperature. Subsequent washes in PBS were performed before the sections were mounted in Vectorshield (Vector Labs, Burlingame, CA, USA). Fluorescent images were captured using confocal microscopy (Olympus FV1000).

### 2.11. Statistical Analysis

Statistical comparisons were conducted using Prism 9 (GraphPad software), with all data presented as mean ± SEM. Student’s *t*-test or one-way ANOVA was applied to assess statistical significance. Post hoc analysis with Tukey’s multiple comparison test was used to evaluate differences between groups in the case of one-way ANOVA. The total fluorescent area was quantified using ImageJ software (1.53a). A *p*-value of less than 0.05 was considered indicative of statistical significance.

## 3. Results

### 3.1. Isolation and Identification of Brain-Derived EVs from SD and DSS Rats

**Figure 1:**
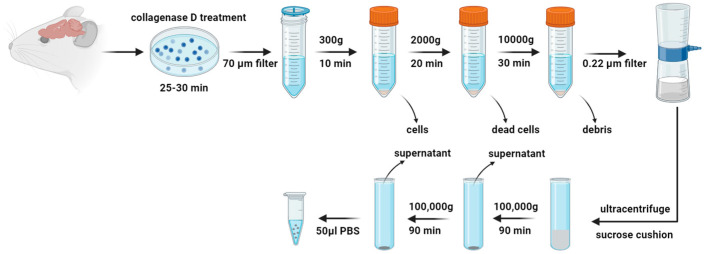
Schematic diagram of brain-derived extracellular vesicle (EV) isolation. Rat brain tissue pieces are first incubated with collagenase D and DNase I to release EVs embedded in the extracellular matrix. A combination of centrifugation, filtration, sucrose cushion ultracentrifugation, and additional ultracentrifugation is performed to isolate brain-derived EVs.

**Figure 2:**
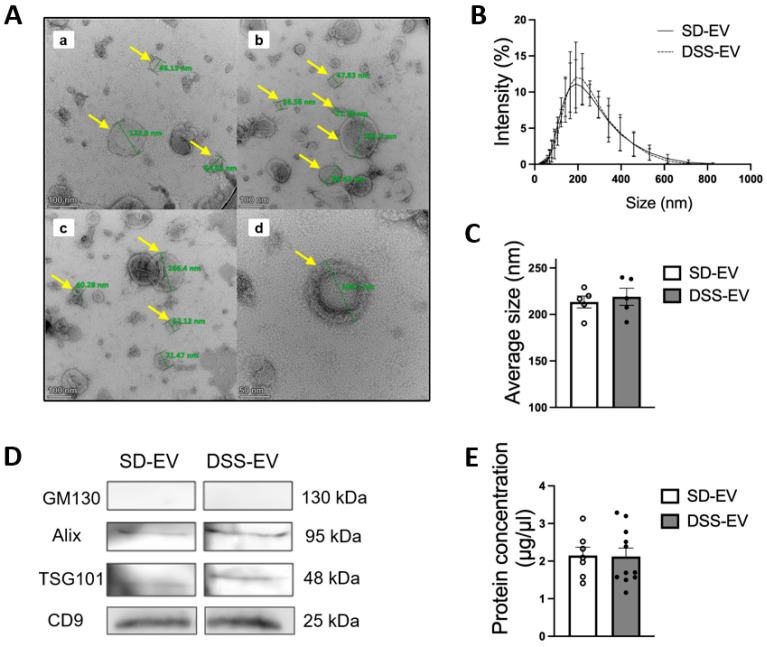
Identification of brain-derived EVs from Sprague Dawley (SD) and Dahl Salt-Sensitive (DSS) rats. (**A**) Representative electron micrograph of brain-derived EVs with transmission electron microscopy; selected EVs are indicated by yellow arrows and their diameters are measured using a green line. The scale bar is 100 nm in (**a**–**c**) and 50 nm in (**d**) (n = 3). (**B**,**C**) Comparison of the size of DSS-EV (dashed line) and SD-EV (solid line) with dynamic light scattering; summary data (**C**) comparing the average size of DSS-EV (n = 5) with SD-EV (n = 5). (**D**) Western blots of EV protein markers (ALIX, TSG101, and CD9) in both groups; GM130 referred as a negative control. (**E**) Protein concentration of brain-derived EVs in SD (n = 7) and DSS (n = 11) rats. Graphs indicate mean ± SEM.

The extraction method for brain EVs from hypertensive DSS rats and their normotensive counterparts, SD rats, is depicted in Figure 1. Utilizing TEM, we confirmed that EVs from both rat groups have a similar range of sizes, 22 to 182 nm, and consist of nanosized vesicles wrapped in a lipid bilayer, as shown in Figure 2A. The DLS method revealed comparable average sizes of EVs from both SD control rats and DSS rats with high salt diets (Figure 2B), with mean diameters of 213.4 ± 6.4 nm for SD-EVs and 218.8 ± 9.2 nm for DSS-EVs, as Figure 2C illustrates. EVs measured via DLS appear larger than observed under the TEM. This discrepancy suggests that the sizing of EVs may be influenced by the specific methods employed. However, a crucial finding from this experiment is that the EV size is comparable between hypertensive and normotensive rats. This finding suggests that hypertension, induced by a high-salt diet in the DSS rats, does not significantly affect the size of the EVs compared to the normotensive SD rats.

Protein concentration measurements of the EVs showed no significant disparity between the two rat groups (Figure 2E; SD-EV: 2.15 ± 0.2 μg/μL vs. DSS-EV: 2.12 ± 0.2 μg/μL). This suggests that the hypertensive state of the donor rat does not impact the total protein content of the EVs. Moreover, a Western blot analysis confirmed the presence of EV markers, ALIX, TSG101, and CD9, in both sets of rats and the absence of the Golgi apparatus protein GM130, which is not associated with EVs, thereby verifying the purity of the EVs extracted from both SD and DSS rat brains (Figure 2D).

We then explored whether the cargos within EVs derived from hypertensive DSS rats and normotensive SD rats have distinct impacts on cellular communication. Specifically, we assessed how these cargos influence the mRNA levels of proinflammatory cytokines (PICs) and the production of ROS in both primary neuronal cultures and the cardiovascular-relevant brain regions of rats.

### 3.2. Brain-Derived EVs from Hypertensive DSS Rats Increase mRNA Levels of PICs and Chemokines in Primary Neuronal Cultures

**Figure 3:**
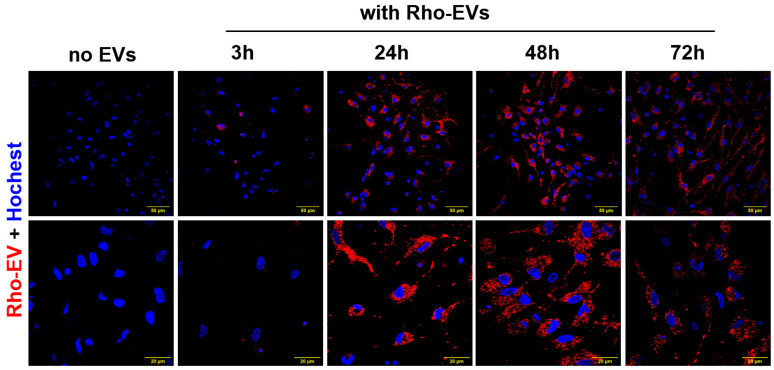
Brain-derived EVs are taken up in primary neuronal cultures. EVs from rat brains were fluorescently labeled (shown in red) and incubated with primary neurons for a series of times—3 h, 24 h, 48 h, and 72 h. Subsequently, labeled EVs were nuclear-stained with Hoechst (shown in blue) and subjected to confocal microscopy (n = 3). The scale bar of images with low magnification is 50 µm and that of images with high magnification is 20 µm.

**Figure 4:**
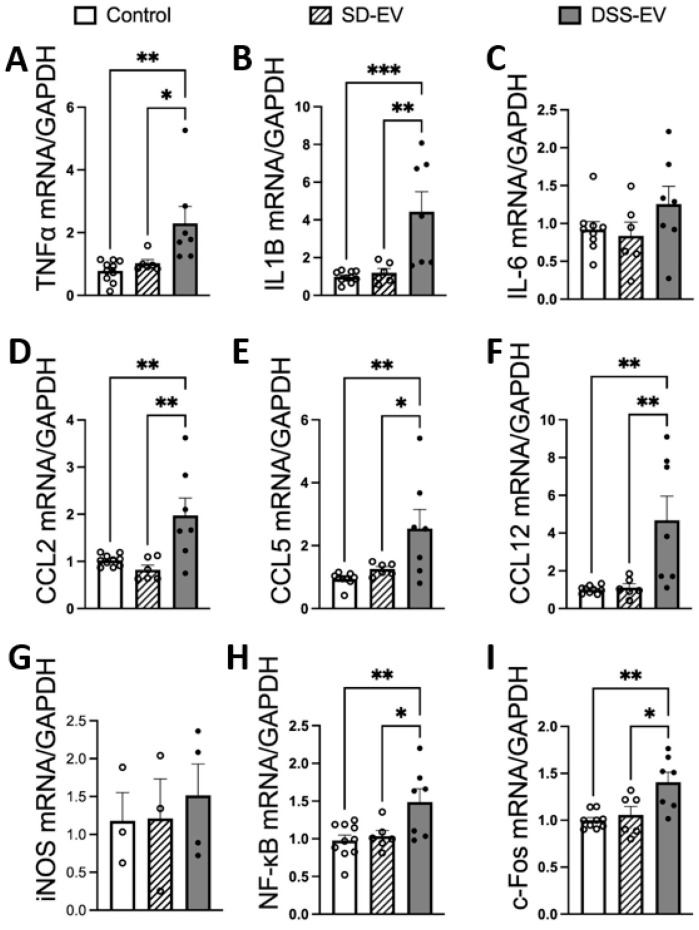
Brain-derived EVs from hypertensive DSS rats increase mRNA levels of inflammatory cytokines, chemokines, NF-kB1, and c-Fos in primary neuronal cultures. Primary neurons were incubated with either DSS-EV, SD-EV, or PBS control for 24 h. The mRNA levels of genes including TNFα (**A**), IL-1β (**B**), IL-6 (**C**), CCL2 (**D**), CCL5 (**E**), CCL12 (**F**), iNOS (**G**), NF-kB (**H**), and c-Fos (**I**) were semi-quantified using real-time PCR and compared among the DSS-EV, SD-EV, and PBS control groups (n = 3–10/group) using the one-way ANOVA test. The mRNA levels were normalized with GAPDH. Dots in the graphs represent individual cell values. Graphs indicate mean ± SEM. * *p* < 0.05, ** *p* < 0.01, *** *p* < 0.001.

Our study provides compelling evidence that brain-derived EVs from hypertensive DSS rats play a crucial role in promoting neuroinflammation, a critical factor in the development of hypertension. Fluorescently labeled EVs were observed to be absorbed by primary rat brain neuronal cultures, with the uptake beginning at 3 h post incubation and reaching a peak at 24 h (Figure 3). This timing provided the framework for subsequent analyses. When treated with EVs from hypertensive rats (DSS-EVs), the neuronal cultures demonstrated a significant upregulation in the mRNA expression of inflammatory markers (Figure 4). Specifically, we observed a 2.3-fold increase in TNFα, a 3.7-fold increase in IL1β, and a 1.4-fold rise in NF-κB, a key regulator of inflammation, compared to the SD-EV-treated group.

This study further revealed that the same treatment with DSS-EVs increased the expression of acute neuronal activation marker c-Fos by 1.3-fold compared to cells treated with EVs from normotensive SD rats. Moreover, the chemokine mRNA levels after DSS-EV exposure showed a consistent pattern of significant elevation: the CCL2 levels rose by 2.4-fold, CCL5 by 2.1-fold, and CCL12 by a striking 4.2-fold, compared to the SD-EV-treated group. These chemokines are known for their role in immune cell mobilization and activation, suggesting an enhanced inflammatory response following treatment with hypertensive rat-derived EVs.

### 3.3. Brain-Derived EVs from Hypertensive DSS Rats Increase mtROS in Primary Neuronal Cultures

**Figure 5:**
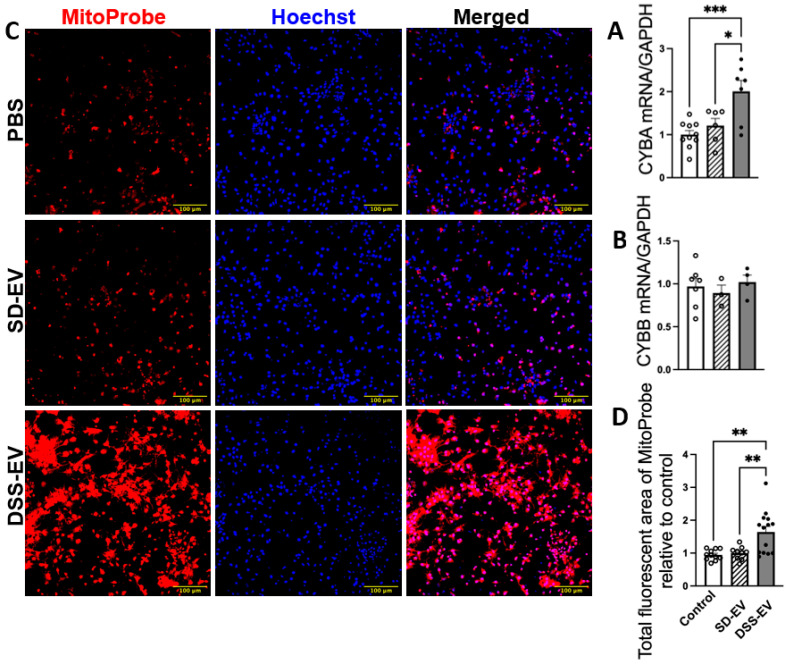
Brain-derived EVs from hypertensive rats increase mtROS in primary neuronal cultures. Primary neurons were incubated with DSS-EV, SD-EV, or PBS control for 24 h. The mRNA levels of CYBA (**A**) and CYBB (**B**) were compared among the DSS-EV, SD-EV, and PBS groups and normalized with GAPDH (n = 3–10/group). (**C**) After 24 h, primary neurons were fixed with 4% PFA and stained with Hoechst (shown in blue) and MitoProbe (shown in red) for 30 min. The scale bar of the images is 100 µm. (**D**) The total fluorescent area of MitoProbe was quantified and normalized to PBS control. Each data point represents the summary data of ROS levels from multiple microscopic views of a cell sample (n = 9–14/group). Graphs indicate mean ± SEM. * *p* < 0.05, ** *p* < 0.01, *** *p* < 0.001.

Our investigation into the connection between oxidative stress and hypertension revealed significant findings. Specifically, we analyzed the effect of DSS-EVs on oxidative stress markers in primary neuronal cultures. The analysis showed that exposure to DSS-EVs resulted in a 1.7-fold increase in the mRNA levels of CYBA, a subunit of NADPH oxidase known for producing ROS (Figure 5A). However, the mRNA levels of another subunit, CYBB, did not significantly change. This suggests a selective regulatory effect of DSS-EVs on components of the NADPH oxidase complex (Figure 5B).

We then measured mtROS production, a key indicator of oxidative stress, using a mitochondria-targeting fluorescent probe, MitoProbe (Figure 5C). The total fluorescent area of mtROS in neurons treated with DSS-EVs was 1.6 times higher than in neurons treated with SD-EVs or PBS (Figure 5D). This indicates that DSS-EVs have a pronounced effect on mtROS production in neuronal cells. This was not observed in neurons treated with SD-EVs, suggesting that the hypertensive state may be linked to increased mitochondrial oxidative stress mediated by EVs.

**Figure 6:**
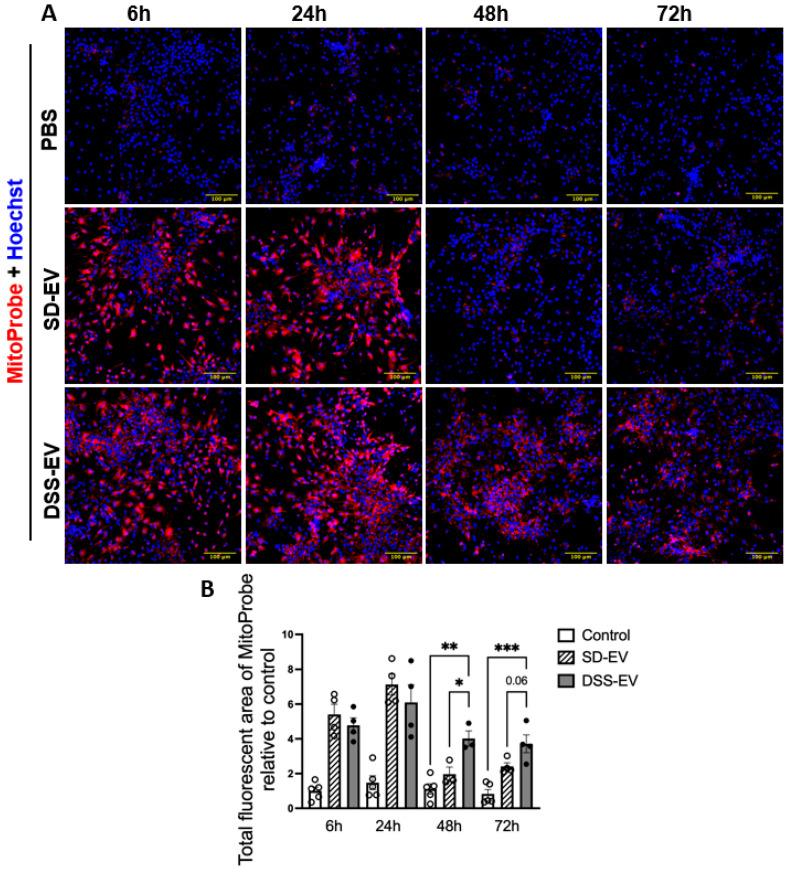
Brain-derived EVs from hypertensive rats increase mtROS in primary neuronal cultures in a time-dependent manner. (**A**) Primary neurons were incubated with DSS-EV, SD-EV, or PBS for a series of times—6 h, 24 h, 48 h, and 72 h. After EV incubation, neurons were immediately stained with Hoechst (shown in blue) and MitoProbe (shown in red) for 30 min. The scale bar of the images is 100 µm. (**B**) The total fluorescent area of MitoProbe was quantified and normalized to the control—6 h group. Each data point represents the summary data of ROS levels from multiple microscopic views of a cell sample (n = 3–5/group). Graphs indicate mean ± SEM. A one-way ANOVA test was performed to compare the fluorescence of different groups treated for the same amount of time. * *p* < 0.05, ** *p* < 0.01, *** *p* < 0.001.

Further time-course analysis showed a time-dependent pattern of ROS production (Figure 6). While both DSS-EVs and SD-EVs induced ROS production initially, after 48 and 72 h, a sharp decline in ROS levels was observed in neurons treated with SD-EVs but not in those exposed to DSS-EVs (Figure 6A). DSS-EV-treated neurons exhibited a 2-fold and 1.6-fold increase in ROS intensity at 48 and 72 h, respectively, compared to SD-EV-treated neurons (Figure 6B). These findings indicate that DSS-EVs maintain a prolonged pro-oxidative effect in primary neurons.

Our data suggest that EVs derived from hypertensive conditions, like those in DSS rats, cause oxidative stress in neurons. This is evidenced by increased mtROS production and the sustained activation of oxidative pathways over time. This study provides compelling evidence that EVs could play a substantial role in the progression of hypertension by perpetuating oxidative stress, highlighting a potential target for therapeutic intervention in hypertensive-related neuronal damage.

### 3.4. ICV Injection of Brain-Derived EVs from Hypertensive DSS Rats Increases mRNA Levels of PICs and Chemokines in the PVN and LT of SD Rats

**Figure 7:**
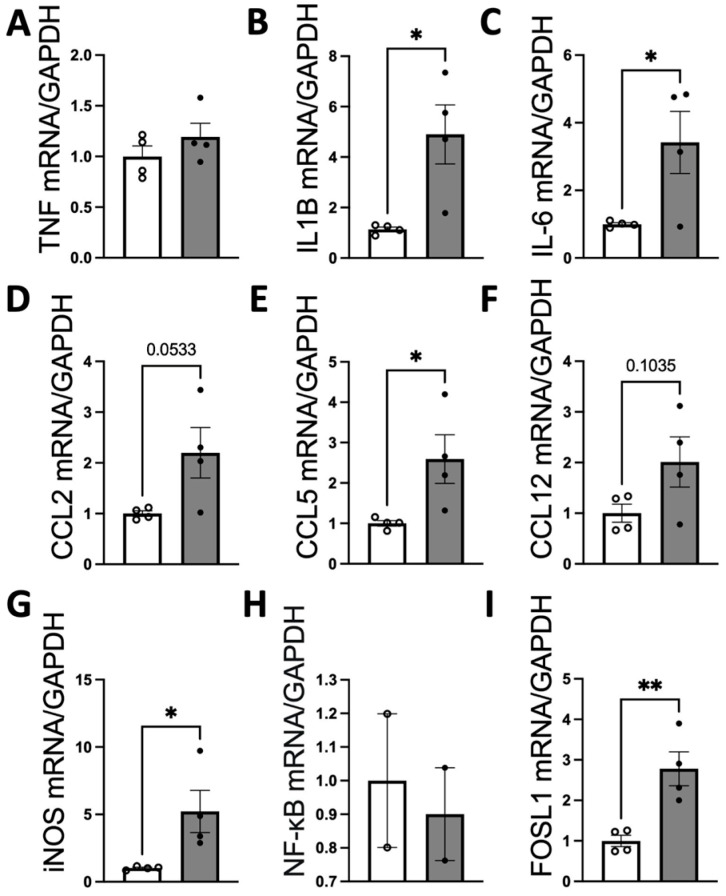
Brain-derived EVs from DSS hypertensive rats increase the mRNA levels of inflammatory cytokines, chemokines, and FOSL1 in the PVN of SD rats. SD rats were administered either DSS-EV (n = 4) or SD-EV (n = 4) into the right lateral ventricle and brain PVN, and punched out 6 h after ICV injection. The PVN mRNA levels of genes including TNFα (**A**), IL-1β (**B**), IL-6 (**C**), CCL2 (**D**), CCL5 (**E**), CCL12 (**F**), iNOS (**G**), NF-kB (**H**), and FOSL1 (**I**) were semi-quantified using real-time PCR and compared between the DSS-EV and SD-EV groups. Dots in the graphs represent the number of individual animals. The mRNA levels were normalized with GAPDH. Graphs indicate mean ± SEM. * *p* < 0.05, ** *p* < 0.01.

**Figure 8:**
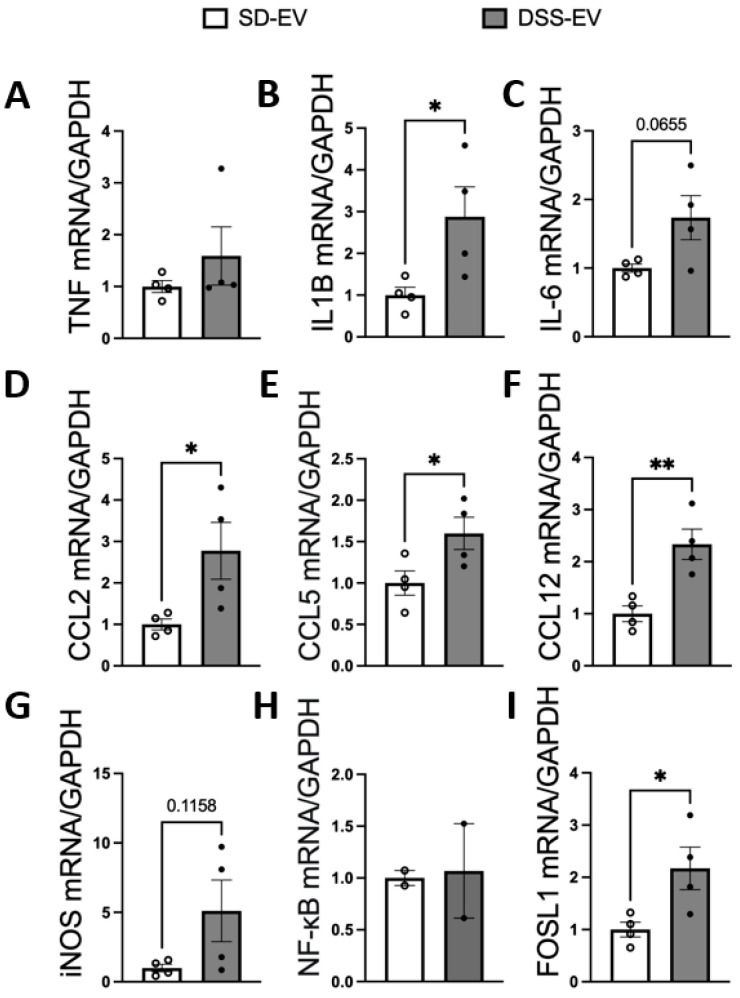
Brain-derived EVs from DSS hypertensive rats increase the mRNA levels of inflammatory cytokines, chemokines, and FOSL1 in the LT of SD rats. SD rats were administered either DSS-EV (n = 4) or SD-EV (n = 4) into the right lateral ventricle and brain LT, and were punched out 6 h after ICV injection. The LT mRNA levels of genes including TNFα (**A**), IL-1β (**B**), IL-6 (**C**), CCL2 (**D**), CCL5 (**E**), CCL12 (**F**), iNOS (**G**), NF-kB (**H**), and FOSL1 (**I**) were semi-quantified using real-time PCR and compared between the DSS-EV and SD-EV groups. Dots in the graphs represent the number of individual animals. The mRNA levels were normalized with GAPDH. Graphs indicate mean ± SEM. * *p* < 0.05, ** *p* < 0.01.

Our results demonstrate that ICV injection of brain-derived EVs from hypertensive DSS rats markedly elevates the mRNA levels of various inflammatory cytokines and chemokines in the PVN and LT of normotensive SD rats (Figure 7 and Figure 8). Specifically, 8 μg of protein-containing DSS-EVs was administered. After 6 h, there was a significant increase in mRNA levels in the PVN for IL1β (4.3-fold), IL-6 (3.4-fold), CCL5 (2.6-fold), iNOS (5.2-fold), and FOSL1 (2.8-fold) when compared to SD-EV injections. Additionally, a trend towards increased mRNA levels of CCL2 and CCL12 was noted. In the LT, a significant upregulation of IL1β (2.8-fold), CCL2 (2.8-fold), CCL5 (1.6-fold), CCL12 (2.3-fold), and FOSL1 (2.1-fold) was observed. These alterations suggest that the injected EVs from hypertensive rats can induce an inflammatory response in brain regions associated with cardiovascular regulation in normotensive rats.

These findings indicate that EVs derived from the brains of hypertensive rats carry inflammatory signatures capable of triggering neuroinflammation and neuronal excitation in otherwise normotensive rats. This discovery, along with the recognized significance of neuroinflammation and neuronal excitation in the development of hypertension, suggests that EVs may play a mediating role in the development and progression of hypertension.

### 3.5. ICV Injection of Brain-Derived EVs from Hypertensive DSS Rats Increases mtROS in PVN and LT of SD Rats

**Figure 9:**
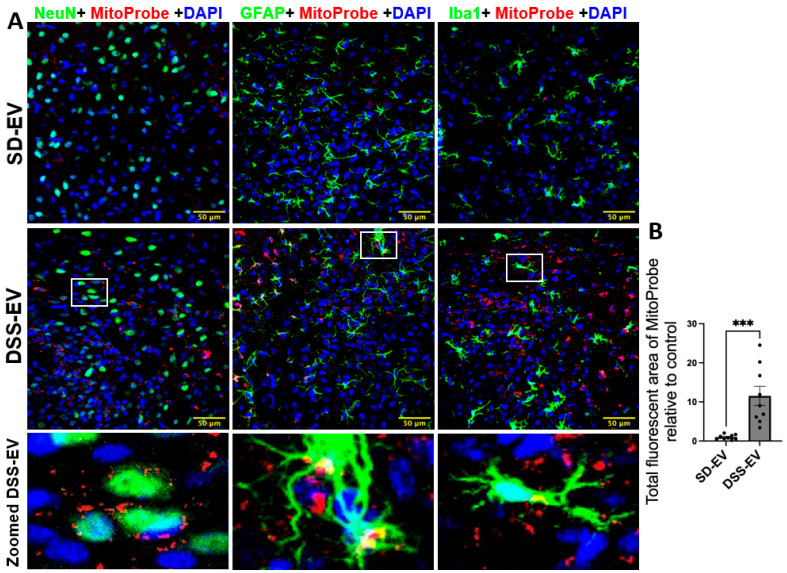
Brain-derived EVs from hypertensive rats increase mtROS in the PVN of SD rats. (**A**) Representative images show PVN ROS levels in different brain cells (NeuN for neurons, GFAP for astrocytes, and Iba1 for microglia; all shown in green) in SD-EV-treated and DSS-EV-treated rats. The scale bar of the images is 50 µm. (**B**) The total fluorescent area of MitoProbe was quantified and normalized to control. Each data point represents the average fluorescent area of multiple microscopic views of a PVN region (n = 9/group). Graphs indicate mean ± SEM. *** *p* < 0.001. (DAPI: 4′,6-diamidino-2-phenylindole dihydrochloride).

**Figure 10:**
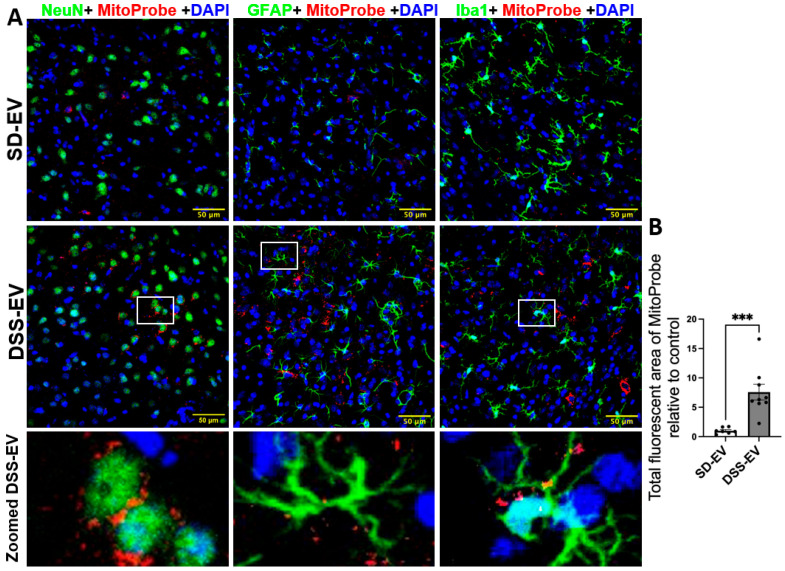
Brain-derived EVs from hypertensive rats increase mtROS in the LT of SD rats. (**A**) Representative images show LT ROS levels in different brain cells in SD-EV-treated and DSS-EV-treated rats. The scale bar of the images is 50 µm. (**B**) The total fluorescent area of MitoProbe was quantified and normalized to control. Each data point represents the average fluorescent area of multiple microscopic views of a LT region (n = 7–9/group). Graphs indicate mean ± SEM. *** *p* < 0.001.

Our results indicate that brain-derived EVs from DSS rats can significantly increase ROS production, leading to oxidative stress within specific PVN and LT regions of the normotensive SD rats. In this experiment, we administered 8 µg of protein-containing EVs from either DSS or SD rats via ICV microinjection into the right lateral ventricle of SD rats. Following this, we injected MitoProbe to assess mtROS levels in the brain. Six hours following EV administration, the rats were euthanized and transcardially perfused with 4% PFA, and their brains were sectioned for mtROS measurement. Confocal imaging demonstrated a notable elevation in mtROS levels in both the PVN and LT regions of rats injected with DSS-EVs, as compared to the control group receiving SD-EV injections (Figure 9A and Figure 10A). Quantitative analysis demonstrated an 11-fold increase in mtROS levels in the PVN and a 7.5-fold increase in the LT, relative to the SD-EV treatment (Figure 9B and Figure 10B).

**Figure 11:**
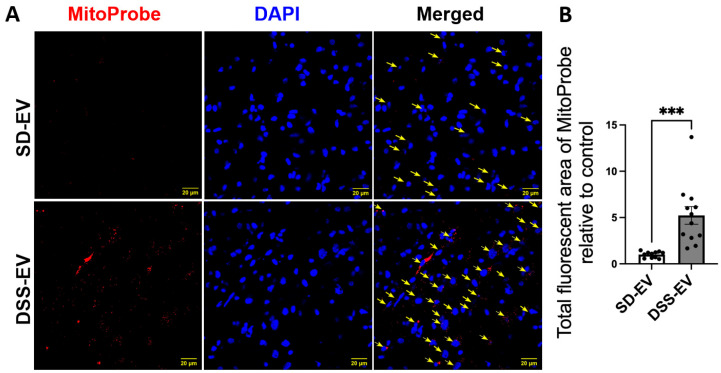
Brain-derived EVs from hypertensive rats increase mtROS in the PVN of SD rats. (**A**) Representative images demonstrate ROS fluorescence in the identified PVN region. Yellow arrows indicate mtROS^+^ cells. The scale bar of the images is 20 µm. (**B**) The total fluorescent area of MitoProbe was quantified and normalized to control. Each data point represents a microscopic view of a PVN region (n = 11–12/group). Graphs indicate mean ± SEM. *** *p* < 0.001.

The analysis of the confocal images further demonstrated that the surge in mtROS was primarily associated with neurons. This was evidenced by the significant overlap between mtROS fluorescence and NeuN staining, a marker for neurons, in both the PVN and LT regions. Additionally, colocalization with GFAP and Iba1 suggested that astrocytes and microglia also contributed to the increased ROS production, albeit to a lesser extent. To validate that the heightened mtROS was indeed a result of DSS-EV introduction and not an artifact of paraformaldehyde fixation, additional rat brains were snap-frozen post EV and MitoProbe injection. The subsequent confocal microscopy of the PVN, after nuclear staining with DAPI, corroborated our findings, showing a significant 5.2-fold increase in ROS production following the DSS-EV treatment compared to SD-EVs (Figure 11).

Our data underscore the role of brain-derived EVs in mediating oxidative stress within the PVN and LT regions under hypertensive conditions. The prominent increase in mtROS levels following the administration of EVs from hypertensive rats suggests that these vesicles could be critical facilitators of oxidative stress. Neurons emerged as the primary cell type affected, with astrocytes and microglia also implicated in the process. These findings bolster the hypothesis that EVs contribute to the pathophysiology of hypertension and may offer novel insights into how hypertension influences brain function, potentially guiding the development of new therapeutic strategies.

### 3.6. Brain-Derived EVs Cross the Blood–Brain Barrier (BBB) and Distribute in the Peripheral Tissues

**Figure 12:**
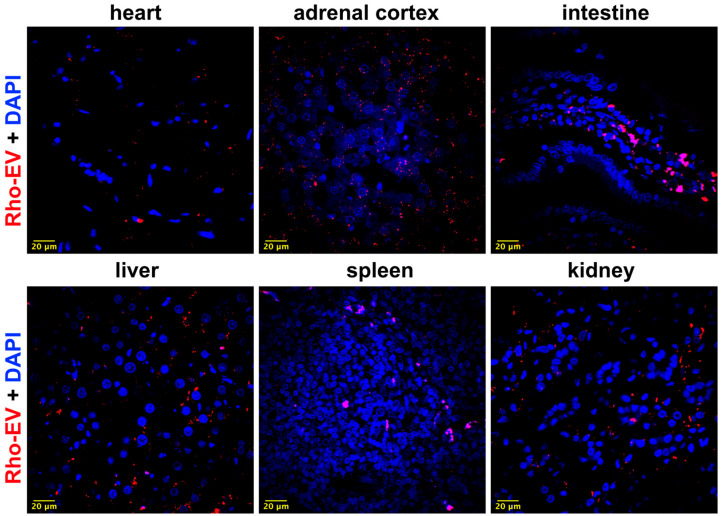
Brain-derived EVs cross the blood–brain barrier. Representative images demonstrate the peripheral biodistribution of fluorescent labeled brain-derived EVs (shown in red). Labeled EVs were injected into the right lateral ventricle of the brain. Tissue sections of the heart, adrenal cortex, intestine, liver, spleen, and kidney were nuclear-stained with DAPI (shown in blue) and subjected to confocal microscopy. The scale bar of the images is 20 µm.

Our abovementioned findings elucidate the influence of brain-derived EVs on neuroinflammation and oxidative stress within the CNS. Building upon this, we aimed to investigate the potential of these brain-derived EVs to cross the BBB and subsequently affect peripheral tissues, thereby broadening their impact beyond the CNS. To assess this, we tracked the distribution of fluorescently labeled EVs across various tissues, including the heart, adrenal gland, intestine, liver, spleen, and kidney, as detailed in Figure 12.

Through confocal microscopy, we observed the presence of labeled EVs in all the aforementioned peripheral tissues. This observation provides compelling evidence that brain-derived EVs can migrate from the CNS to peripheral areas. The detection of these EVs in peripheral tissues underscores their potential role in systemic physiological or pathological processes, extending the known effects of EVs beyond their origin in the brain.

## 4. Discussion

Our current study embarked on a quest to decode the influence of brain-derived EVs on the progression of hypertension, focusing on their ability to modulate neuroinflammation and the generation of ROS in primary neuronal cultures and brain regions critical for cardiovascular regulation. The investigation unraveled three pivotal findings:

(1) EVs isolated from the brains of hypertensive DSS rats were identified to significantly increase PIC levels, such as TNF-α and IL-1β, in cultured neurons. This suggests that the EVs can carry and transfer inflammatory signals, potentially leading to a heightened inflammatory state in the recipient cells. The activation of NF-κB signaling pathways and the elevation of chemokines like CCL2, CCL5, and CCL12, as well as the amplification of neuronal activity evidenced by the c-Fos marker, were also noted. Furthermore, an increase in ROS within these neurons was observed, hinting at the pro-oxidative effects of the EVs.
(2) The administration of EVs from hypertensive rats to normotensive SD rats resulted in similar inflammatory and oxidative responses within the PVN and LT—brain regions that are instrumental in managing cardiovascular functions. This reaction was characterized by elevated levels of proinflammatory markers, neuronal activity as indicated by Fosl1, and mitochondrial ROS production.
(3) A pronounced rise in ROS production, specifically in neurons located within the PVN and LT, was induced by the hypertensive rat brain-derived EVs, highlighting the targeted nature of EV-related alterations within these crucial neural structures.

Our findings confirm the essential role of EVs, such as exosomes and larger vesicles, in cell-to-cell communication. These EVs act as carriers for biological messages that can change how cells function, highlighting their possible involvement in causing hypertension. By comparing EVs from both normotensive and hypertensive rats, we aim to understand how they may contribute to complications associated with hypertension.

To ensure the quality of our EV samples, we relied on recognized EV markers. Our analysis indicated that the total protein levels in EVs from both normotensive and hypertensive rats were similar, suggesting that the protein quantity of EVs is not affected by hypertension. We confirmed the purity of our EV samples by detecting specific EV markers (ALIX, TSG101, and CD9) and ensuring the absence of GM130, a non-EV marker. This suggests that the essential characteristics of EVs are maintained irrespective of the donor’s BP.

Initially, we chose markers such as ALIX, TSG101, and CD9 for their well-established presence in EV research, which allows us to align our work with previous studies. However, incorporating neuron-specific markers like NCAM will likely yield a deeper insight into the specific neural contributions to EV profiles.

Looking ahead, we plan to include neuron-specific markers, like NCAM [33], in our studies to confirm the neural origin of EVs and investigate changes in EV content related to hypertension. This could enhance our understanding of the influence of neural-derived EVs on hypertension and may lead to the discovery of new biomarkers or therapeutic targets.

We acknowledge the value of having a control group of DSS rats on a low- or standard salt diet to directly compare how increased salt intake affects neuroinflammation and hypertension. However, we chose SD rats as our normotensive control group instead of DSS rats on a normal salt diet to emphasize the impact of a genetic tendency towards salt sensitivity on the results. This choice is backed by our previous research, which showed that DSS rats on a high-salt diet had significantly higher mean arterial BP (MAP) and neuroinflammation markers than those on a normal salt diet, illustrating a genetic inclination that intensifies the response to high salt levels [11].

Including SD rats with their standard diet as normotensive controls establishes a reference point against a contrasting genetic background. By comparing these different genetic profiles, we aimed to shed light on the varied reactions to a high-salt diet between DSS rats, which are genetically salt-sensitive, and SD rats, which are not. Our research found that a high salt intake drastically raised MAP and caused neuroinflammation in DSS rats, but such significant changes were not seen in SD rats given the same diet. This difference highlights the importance of genetic factors in salt sensitivity and its consequent impact on BP and neuroinflammation.

Moreover, by examining the different responses between DSS and SD rats, we gain crucial insights into the physiological and molecular underpinnings of salt-sensitive hypertension and its neuroinflammatory effects. This comparison is particularly valuable for exploring how genetic predispositions and environmental influences, such as diet, interplay in the development of hypertension. Such nuances might remain hidden if our analysis were limited to comparing only DSS rats subjected to varying salt intakes.

In line with this, our current research delves deeper into the function of EVs in the central nervous system of hypertensive rats, focusing on how they might influence primary neuronal cultures and potentially drive neuroinflammatory processes. Neuroinflammation is characterized by elevated levels of PICs, which are linked to several health conditions, including hypertension. Our findings indicate that EVs from hypertensive rats increase the mRNA levels of inflammatory markers such as TNFα and IL1β and the activation of the transcription factor NF-κB in neuron cultures. The activation of NF-κB, a key regulator of many genes involved in inflammation, suggests initiating an inflammatory state within these cells.

Our previous research established that TNFα could intensify the inflammatory response in neurons [34], a finding reinforced by our current study. Neurons are thus implicated as both sources and targets of neuroinflammation in hypertensive conditions, responding to external hypertensive stimuli, in this case, brain-derived EVs. Moreover, EVs have been known to carry or present cytokines on their surface, including TNFα, IL1β, and IL6 [35]. This aligns with findings in clinical settings, such as in patients with myocardial infarction, where EV-associated cytokines have shown dysregulation [36].

While our current work did not determine the exact source of central cytokines, further research is necessary to elaborate. The primary neuronal cultures were derived from various brain regions, including the hypothalamus and cortex. To pinpoint the brain areas most affected by EVs, we examined the inflammatory responses in two hypertension-relevant regions, the PVN and LT, after introducing EVs intracranially. Consistently, we observed that EVs from hypertensive rats induced a significant increase, or at least a rising trend, in cytokine and chemokine levels in these regions compared to controls. This aligns with previous observations that a high-salt diet increases cerebrospinal fluid sodium levels and that certain cytokines are elevated in hypertensive conditions [11]. Notably, injections of PICs into the PVN have been shown to raise BP and SNA, implicating these cytokines in the hypertension development [37].

Our present study discovered a marked increase in IL1β and a notable rising trend in IL6 in the LT, characterized by its highly permeable blood–brain barrier. This suggests that neuroinflammation in the LT during hypertension may be mediated, at least in part, by brain-derived EVs. Given the LT’s susceptibility to circulating cytokines, it is plausible that these cytokines could affect its functions, a hypothesis supported by models indicating that the LT’s neural response to cytokines contributes to hypertension development [38,39]. These findings suggest that brain-derived EVs may serve as conduits for neuroinflammation, potentially amplifying the inflammatory environment within the brain, which is associated with the onset and progression of hypertension.

Our investigation expanded to the impact of EVs on chemokine expression, where we discovered pronounced increases in the mRNA levels of CCL2, CCL5, and CCL12 in primary neuronal cultures exposed to EVs from hypertensive DSS rats. Notably, in brain regions such as the PVN and the LT, we detected either significant enhancements or an upward trend in the mRNA levels of these chemokines when SD rats received treatments with EVs sourced from hypertensive DSS counterparts. These findings are pivotal due to the established role of these chemokines in hypertension.

The chemokine CCL2 is crucial in guiding immune cells like neutrophils, monocytes, and T cells to inflammation sites by interacting with specific chemokine receptors. The link between high CCL2 levels and the severity of hypertension-related organ damage has been supported by correlations found in human studies [40]. Furthermore, experiments blocking CCR2, the receptor for both CCL2 and CCL12, demonstrated a reduction in BP in animal models of hypertension [41].

CCL5 is another critical chemokine, serving as a strong attractant for monocytes and T cells. Increased levels of CCL5 have been documented in both the central nervous system and peripheral organs such as the kidneys in hypertensive rats, implying a systemic response that could exacerbate hypertension [42,43,44].

The elevated mRNA levels of CCL2 in neurons treated with EVs from hypertensive rats suggest that these vesicles may carry factors that induce an inflammatory response. Since CCL2 is involved in recruiting inflammatory cells, its increased expression could lead to enhanced inflammation within the brain, contributing to the pathophysiology of hypertension. The fact that higher CCL2 levels are associated with more severe organ damage in humans with hypertension underscores the clinical relevance of our findings.

The heightened expression of CCL5 in response to EVs from hypertensive rats highlights a potential mechanism by which hypertension can alter immune cell migration and infiltration in the brain and other organs. Given that CCL5 expression is upregulated in hypertensive rats’ central nervous system and kidneys, it is plausible that this chemokine could contribute to maintaining and progressing high BP through its chemotactic activities. The observed increase in CCL12 mRNA and the known effects of blocking CCR2 present a strong case for these molecules as therapeutic targets. By interfering with CCR2, the EV-mediated propagation of hypertension could be mitigated, reducing BP and associated damage.

Our observations indicate a substantial role for brain-derived EVs in mediating chemokine-driven inflammatory responses, implicating them in the recruitment and activation of immune cells that potentially exacerbate neuroinflammation. This could have far-reaching implications for understanding the pathogenesis of hypertension and open the door to novel therapeutic strategies targeting EVs and their inflammatory cargo.

Our research further delves into the contribution of EVs to ROS generation. ROS can eclipse the body’s antioxidant defenses when produced excessively, prompting oxidative stress—a pivotal factor in the onset of hypertension [45]. Our findings highlight that exposure to EVs from the brains of hypertensive DSS rats significantly escalates CYBA mRNA levels in primary neuronal cultures. This suggests that EVs may activate oxidative stress by engaging the NADPH oxidase system, a known pathway for ROS production. While mitochondria are recognized as the primary sites for ROS generation, our assays of mtROS through fluorescence intensity reinforce the premise that DSS-EVs amplify mtROS production.

To substantiate the link between EVs and oxidative stress in vivo, we administered ICV injections of DSS-EVs into normotensive SD rats. Post injection, we observed a pronounced rise in iNOS mRNA within the PVN and a surge in mtROS fluorescence in both the PVN and LT. Our data pinpoint neurons as the predominant ROS generators in response to EV exposure. This is a reasonable observation considering that neurons, with their high metabolic demand, possess an abundance of mitochondria, which, under stress conditions, can leak electrons that react with oxygen to form ROS. Therefore, neuronal cells are particularly susceptible to shifts in redox states and can significantly contribute to ROS production. This discovery is crucial because it directly connects neuroinflammation, oxidative stress, and hypertension. Both neuroinflammation and oxidative stress are known to amplify the SNA, which is a major driving force in the development of hypertension. ROS and inflammatory mediators can disturb neurotransmitter equilibrium and autonomic regulation, increasing the SNA [20,46]. In line with these findings, our experiments showed that treatment with DSS-EVs increased the expression of c-Fos, a marker of neuronal activation, in primary neuron cultures. We also noted higher levels of FOSL1, another indication of neuronal activity, in the PVN and LT regions. Such neuronal activation is a precursor to raised BP and the advancement of hypertension.

Our findings suggest that EVs derived from normotensive rats have a dual impact on cellular processes. Initially, these EVs may cause an increase in mtROS, likely because they carry mitochondrial fragments, which are known to be sources of ROS. However, the same EVs may also contain specific cargos, such as superoxide dismutase (SOD) and catalase, which could counteract ROS. Furthermore, these EVs may promote the expression of antioxidant genes, resulting in a decrease in oxidative stress and an increase in antioxidant defenses in neural cells.

In addition to this, studies have shown that EVs can encapsulate mitochondrial DNA (mtDNA) and mitochondrial proteins, both of which are protected within the vesicles [47]. This selective encapsulation and transportation of mitochondrial elements by EVs are critical for intercellular communication, influencing various processes such as energy metabolism, immune response, and recovery from injury [47]. The imbalance of these processes may be evident in hypertensive rats, where EVs could contribute to sustained oxidative stress, potentially due to a lack of antioxidative substances or the presence of pro-oxidative elements. This concept is partly supported by findings in heart failure models [48], where EVs carry microRNAs that inhibit antioxidant pathways, leading to a redox imbalance.

We have observed that after 24 h, mtROS levels in cells exposed to EVs from normotensive rats return to baseline, suggesting a self-regulating mechanism within the EVs. In contrast, EVs from hypertensive rats do not show this return to balance, implying a deficiency in antioxidative capacity or an overabundance of oxidative agents.

Recognizing that mild to moderate ROS levels can benefit cellular adaptation, our study still needed to explore how different doses of EVs might influence ROS production. Since the biological effects of EVs, such as on angiogenesis, are dose-dependent, the dosage could be a critical factor in the observed cellular outcomes [49,50].

Our recent findings highlight that EV exposure is a determinant in cellular outcomes, as EVs significantly influence mtROS production. Understanding the specific EV components responsible for the initial mtROS increase and subsequent moderation is crucial. Therefore, our next research phase will focus on identifying these components and examining the dose-dependent effects of EVs on ROS production. This will advance our knowledge of EVs’ roles in cellular functions and guide the development of EV-based therapies.

Moving forward, we will dissect the components within EVs that contribute to both ROS increase and neutralization. Additionally, we will investigate the dose-dependent effects of EVs on ROS production to fully understand their therapeutic potential and ensure the safety of EV-based interventions.

Research has shown that cytokines, which are signaling proteins in the CNS, can pass into the bloodstream. This was notably demonstrated by Chen et al. [51,52], who found that after injecting cytokines such as IL-6 and TNF-α directly into the brain’s ventricles, a substantial amount of these cytokines could be detected in the circulatory system. These findings imply that the CNS might not only be a source of cytokines found in blood but also a regulator of peripheral metabolic, endocrine, and immune functions.

Further studies have indicated that specific cytokines, including various forms of interleukin (IL-1α, IL-1β, and IL-1ra) and TNFα, can cross the BBB through specific transport mechanisms, potentially affecting brain function [53,54]. This bidirectional exchange of cytokines between the CNS and the bloodstream suggests a pathway through which blood-borne cytokines may impact brain activities [53,54,55,56].

Our present study explored the concept that brain-derived EVs can traverse the BBB. By marking these EVs with a mitochondrial-targeted fluorescent dye [29], we could follow their journey to remote organs, such as the heart and kidneys in rats. This movement was verified by confocal microscopy, providing new insights into how substances originating in the brain might systematically influence other organs, particularly the kidneys.

The ability to track EVs moving across the BBB represents a significant step in our research. It establishes a foundation for future studies examining the involvement of EVs in CNS disorders and their impact on peripheral organs. This work significantly expands our knowledge of the complex communication pathways between the brain and other body parts.

Our research is an exploratory investigation into how EVs from rats with hypertension impact inflammation and oxidative stress in nerve cells and brain regions responsible for cardiovascular regulation. This study plays a pivotal role in uncovering the potential involvement of EVs in the initiation of hypertension. A notable limitation in this work was the need for direct BP measurements following the introduction of EVs, which we aim to address in future research.

Furthermore, the renin–angiotensin system is a pivotal hormonal system governing BP regulation. Angiotensin II (AngII), a potent vasoconstrictive peptide within this system, is known to elevate BP by binding with its type I receptor [57]. Therefore, our further research will investigate the direct impact of EVs derived from hypertensive donors on both BP and AngII hormone levels in the brain and circulation of normotensive recipients. These studies are anticipated to yield substantial evidence, reinforcing the hypothesis that brain-derived EVs may play a role in the development of hypertension.

In summary, our research elucidates the crucial role of brain-derived EVs on inflammation and oxidative stress, highlighting their potential contribution to the development of hypertension and peripheral diseases. Using EVs as biomarkers or therapeutic agents [58] in treating neurological disorders opens up novel approaches to managing a spectrum of diseases, extending from neurodegenerative to systemic illnesses. By incorporating specific neural markers in future research, we anticipate a deeper understanding of the origins and roles of brain-derived EVs. Moreover, our methods for detecting EVs in the bloodstream could revolutionize both research and clinical applications of EVs, heralding future breakthroughs in this domain.

## 5. Conclusions

**Figure 13:**
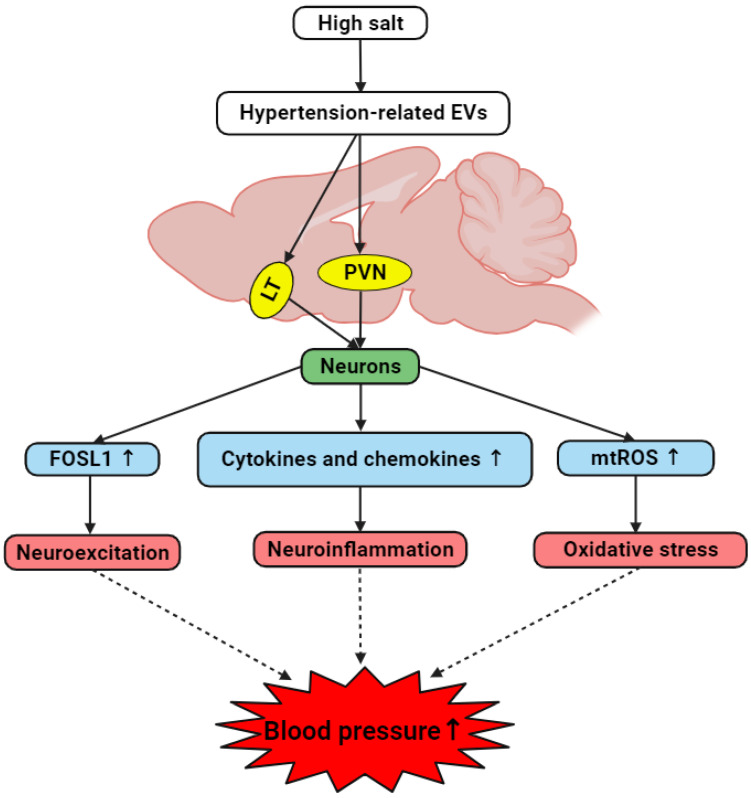
The hypothesized interplay between brain-derived EVs and hypertension development in hypertensive DSS rats. A high-salt diet induces the generation of brain-derived EVs containing hypertension-associated factors. These hypertension-related EVs target cardiovascular regions including LT and PVN, and trigger neurons to elevate the levels of FOSL1, cytokines, chemokines, and mitochondrial ROS. Consequently, this results in neuroexcitation, neuroinflammation, and oxidative stress, collectively contributing to the onset and/or progression of hypertension.

Our present study has found that brain-derived EVs from hypertensive rats may influence the onset and progression of hypertension by modulating neuroinflammation and ROS production within key cardiovascular regions of the brain and primary neuronal cultures. The current study has identified a notable increase in inflammatory markers and neuronal activity indicators in response to hypertensive EVs, suggesting that these vesicles may serve as critical facilitators of the inflammatory processes associated with hypertension. Our work provides a valuable perspective on the role of EVs as carriers of pathological signals in hypertensive conditions. By demonstrating that EVs derived from hypertensive brains can elevate cytokine and chemokine levels, increase markers of neuronal activity, and promote excessive mtROS production, we have established a potential mechanistic link between EV-mediated intercellular communication and hypertension (Figure 13).

The implications of these findings are profound, as they highlight the potential of targeting EVs to alleviate the neurological and cardiovascular complications of hypertension. Our results underscore the importance of further exploring the biological significance of EVs in hypertension, which could lead to innovative therapeutic approaches that address the root causes of this condition. Future research in this domain is poised to refine our understanding of EV-mediated pathways in hypertension, paving the way for novel interventions that could transform the management of this prevalent and debilitating disease.

## 6. Limitations

Our study sheds light on the potential role of EVs from hypertensive DSS rats in promoting neuroinflammation and an increase in ROS production, which may contribute to the development of hypertension. However, we encountered several limitations that warrant further investigation. For instance, the use of primary neuronal cultures from diverse brain regions did not allow for the identification of specific regional responses to EV exposure. Additionally, the study did not delve into the origins of central cytokines, underscoring the need for more detailed research to elucidate the mechanisms by which EVs influence BP.

In this research, our initial focus was on male rats to limit biological variability. Recognizing the significance of including both genders, we plan to expand our studies to encompass female rats. This expansion, adhering to NIH guidelines, aims to enrich our research’s scope and ensure that the insights we gain on the role of extracellular vesicles in hypertension are comprehensive, addressing both male and female physiology.

Moreover, our current investigation centered on assessing the role of EVs in modulating inflammation and oxidative stress without directly measuring BP following EV administration. Future research will incorporate this measurement to address this gap and offer a fuller picture of how EVs contribute to hypertension.

Lastly, our study did not examine the long-term effects of EV exposure nor investigate potential interventions to modulate EV-mediated effects. Future endeavors will explore these areas to deepen our understanding of the therapeutic potential of targeting EVs in hypertension, thereby paving the way for innovative treatment strategies.

## Data Availability

The data presented in this study are available on request from the corresponding author due to privacy.

## Supplementary Materials

The following supporting information can be downloaded at: https://www.mdpi.com/article/10.3390/antiox13030328/s1, Table S1: Primer sequences, primary antibodies, and reagents.
