# Supplementary material for: Induction of Neuroinflammation and Brain Oxidative Stress by Brain-Derived Extracellular Vesicles from Hypertensive Rats
Source: Antioxidants (Basel). 2024 Mar 7;13(3):328. doi: 10.3390/antiox13030328 (PMC10967780; doi:10.3390/antiox13030328)
Supplement: Supplementary file 1 [file antioxidants-13-00328-s001.zip › antioxidants-2863326-supplementary.pdf]

Supplementary materials

Table S1. Primers, gene accession numbers, antibodies, and reagents

**Primers**	**Assay ID**	**Accession No.**
Tumor Necrosis Factor alpha (TNFα)	Rn99999017_m1	NM_012675.3
Interleukin-1 beta (IL-1β)	Rn00580432_m1	NM_031512.2
Interleukin 6 (IL-6)	Rn01410330_m1	NM_012589.2
C-C Motif Chemokine Ligand 2 (CCL2)	Rn00580555_m1	NM_031530.1
C-C Motif Chemokine Ligand 5 (CCL5)	Rn00579590_m1	NM_031116.3
C-C Motif Chemokine Ligand 2 (CCL12)	Rn01464638_m1	NM_001105822.1
Nitric Oxide Synthase, Inducible (iNOS)	Rn00561646_m1	NM_012611.3
Nuclear factor of kappa light polypeptide gene enhancer in B-cells 1 (NF-kB)	Rn01399583_m1	NM_001276711.1
FBJ osteosarcoma oncogene (c-Fos)	Rn02396759_m1	NM_022197.2
Fos-like antigen 1 (FOSL1)	Rn00564119_m1	NM_012953.1
Cytochrome b-245, alpha polypeptide (CYBA)	Rn00577357_m1	NM_024160.1
Cytochrome b-245, beta polypeptide (CYBB)	Rn00576710_m1	NM_023965.1
Glyceraldehyde-3-phosphate dehydrogenase (GAPDH)	Rn01775763_g1	NM_017008.4
**Primary Antibodies**	**Accession No.**	**Working concentration**
Anti-GM130 (Golgi matrix protein 130 kD, GM130)	sc-55591	1:200
Anti-Alix (ALG-2 interacting protein X, Alix)	sc-53540	1:200
Anti-TSG101 (tumor susceptibility 101, TSG101)	sc-7964	1:200
Anti-CD9 (CD9 molecule, CD9)	98327	1:1000
Anti-NeuN (Neuronal nuclei, NeuN)	24307	1:300
Anti-GFAP (Glial fibrillary acidic protein, GFAP)	3670	1:300
Anti-Iba1 (Ionized calcium binding adaptor molecule 1, Iba1)	019-19741	1:500
**Reagents**	**Accession No.**	**Source**
Collagenase D	11088866001	Sigma-Aldrich
DNase I	11284932001	Sigma-Aldrich
Protease and Phosphatase Inhibitor Cocktail	PPC1010	Sigma-Aldrich

Hibernate™-E Medium	A1247601	ThermoFisher Scientific
RNeasy Mini Kit (250)	74106	QIAGEN
iScript™ cDNA Synthesis Kit	1708891	Bio-Rad Laboratories
TaqMan™ Fast Advanced Master Mix for qPCR	4444964	ThermoFisher Scientific
RIPA Buffer (10X)	9806	Cell Signaling
Phenylmethanesulfonyl Fluoride (PMSF)	8553	Cell Signaling
Protein Standard II	5000007	BIO-RAD
Bradford Reagent	B6916	Sigma-Aldrich
10x Tris/Glycine/SDS Buffer	1610772	BIO-RAD
10x Tris Buffered Saline (TBS)	1706435	BIO-RAD
SuperSignal™ West Dura Extended Duration Substrate	34075	ThermoFisher Scientific
VECTASHIELD® HardSet™ Antifade Mounting Medium with DAPI	H-1500-10	Vector Labs
Hoechst	62249	ThermoFisher Scientific
Donkey anti-rabbit IgG, Alexa Fluor™ 488	A32790	ThermoFisher Scientific
Donkey anti-mouse IgG, Alexa Fluor™ 488	A-21202	ThermoFisher Scientific
Rabbit IgG HRP Linked Whole Ab	NA934	Sigma-Aldrich
Mouse IgG HRP Linked Whole Ab	NA931	Sigma-Aldrich
